# Glycan heterogeneity on gold nanoparticles increases lectin discrimination capacity in label-free multiplexed bioassays†

**DOI:** 10.1039/c6an00549g

**Published:** 2016-05-16

**Authors:** Lucienne Otten, Denise Vlachou, Sarah-Jane Richards, Matthew I. Gibson

**Affiliations:** a Department of Chemistry, University of Warwick, Coventry, CV4 7AL, UK; b Warwick Medical School, University of Warwick, Coventry, CV4 7AL, UK

## Abstract

The development of new analytical tools as point-of-care biosensors is crucial to combat the spread of infectious diseases, especially in the context of drug-resistant organisms, or to detect biological warfare agents. Glycan/lectin interactions drive a wide range of recognition and signal transduction processes within nature and are often the first site of adhesion/recognition during infection making them appealing targets for biosensors. Glycosylated gold nanoparticles have been developed that change colour from red to blue upon interaction with carbohydrate-binding proteins and may find use as biosensors, but are limited by the inherent promiscuity of some of these interactions. Here we mimic the natural heterogeneity of cell-surface glycans by displaying mixed monolayers of glycans on the surface of gold nanoparticles. These are then used in a multiplexed, label-free bioassay to create ‘barcodes’ which describe the lectin based on its binding profile. The increased information content encoded by using complex mixtures of a few sugars, rather than increased numbers of different sugars makes this approach both scalable and accessible. These nanoparticles show increased lectin identification power at a range of lectin concentrations, relative to single-channel sensors. It was also found that some information about the concentration of the lectins can be extracted, all from just a simple colour change, taking this technology closer to being a realistic biosensor.

## Introduction

Protein–carbohydrate interactions are crucial for many biological processes including both passive and innate immunity, cell–cell communication, protein folding and fertilsation.^1^ Their prevelance in essential processes means they are exploited by pathogens in their initial adhesion step prior to infection. Carbohydrate-binding proteins (known as lectins) mediate these adhesion steps. Lectin binding is determined by a combination of factors including carbohydrate branching, stereochemistry, and chemical functionality.^2^,^3^ Despite their role in signalling, these binding events tend to be weak (*K*_d_ = 10^3^–10^6^ M^−1^). This is circumvented in nature by the presentation of multiple copies of the target carbohydrate which gives rise to an increase in binding affinity that is greater than the linear sum of the binding to the individual carbohydrates (known as the ‘cluster glycoside effect’).^4^–^6^ This increased binding affinity due to multivalency has been widely exploited to create synthetic glycomimetics, such as glycopolymers,^7^–^12^ glycoparticles^13^–^16^ and glyco-surfaces.^17^ However, many lectins show highly specific binding to oligosaccharides but show much more promiscuous binding characteristics on a mono- and disaccharide level^18^ meaning a balance must be struck between synthetic accessibility and observed specificity when integrating these into materials.

Interactions between lectins on the host cell surface and carbohydrate epitopes on the microbe/virus/toxin can initiate infection. For example, ricin is a toxic lectin that presents a potential security threat. It is a lethal, type 2 ribosome-inactivating protein found in the castor bean plant *Ricinus communis*. It is an A–B toxin whereby its B-chain adheres to terminal galactose residues on mammalian cell surfaces, facilitating the delivery of the toxic A-chain into the cytosol of the cell. The A-chain catalyses the hydrolytic cleavage of a single base from eukaryotic ribosomal RNA, leading to a shutdown in protein synthesis and ultimately cell death.^19^ Ricin is several thousand times more toxic to man than cyanide, with the median lethal dose (LD_50_) for an adult estimated to be around 22 μg per kg of body weight (<1.8 mg for an average adult). Therefore, rapid detection of various lectin types is required, not only for diagnosis but also for developing effective therapeutics. However, glycans are inherently complex and it is observed that lectins are capable of binding a range of related carbohydrate structures to varying extents, further complicating the challenge of assigning protein–carbohydrate interactions.^20^ Carbohydrate microarrays have been developed to aid in the identification of protein–carbohydrate interactions as new drug targets,^21^–^24^ however, they are difficult to construct and require a protein-labelling step which results in heterogeneity, synthetic complexity and is not a truly native assay.^25^ Therefore the development of fast, label-free, easy and inexpensive sensors for the analysis of carbohydrate–protein interactions is highly desirable.

Glyconanoparticles, with multivalent presentation of the glycans, are rapidly emerging as biosensors, in imaging and in therapeutics^26^ and are thus a good biomimetic model of carbohydrate presentation at the cell surface. Gold nanoparticles (AuNPs) exhibit characteristic optical properties that are dependant on surface plasmon resonance, which arises due to collective oscillations of the conduction electrons. Monodisperse AuNPs between 10 and 80 nm appear red in colour, but as the interparticle distance decreases coupling between the dipole–dipole interactions between the plasmons of neighbouring particles leads to a broadening and a shift to longer wavelengths of the surface plasmon absorption band, resulting in the AuNPs appearing blue. The colourimetric change can be detected spectrophotometrically and visually^27^ and has been used to probe biomolecules such as proteins,^28^,^29^ peptides,^30^ antibodies,^31^–^36^ and DNA^37^,^38^ without needing labelled proteins. The architecture of the presentation of the glycans on the particle surface has a huge impact on the output of these assays. Otsuka *et al*. found that, for 9 nm particles bearing lactose residues, more than 20% lactose was required for aggregation to occur in the presence of *Ricinus communis* agglutinin (RCA_120_).^39^ Schofield *et al*.^40^ described AuNPs stabilised with 9-mercapto-3,6-diaoxaoctyl-β-d-galactoside and a thiolated triethylene glycol derivative as a dilutant to investigate the effect of carbohydrate surface coverage. Interestingly, it was found that a 70% coverage of galactose was optimum for RCA^120^ detection, they also noted that a short linker between the particle and the pendant galactose gave greater aggregation, however, the longer chain gave a more stable sensing system. The same was carried out for Concanavalin A (Con A), it was found that 100% carbohydrate coverage was optimum for this protein.

The above examples show that controlling glycan presentation can significantly improve specificity and affinity. However, to discriminate between a large number of lectins with similar specificities either complex glycans, or multiplexed assays are often needed. Jayawardena *et al*.^41^,^42^ used a range of glycosylated nanoparticles to distinguish between a range of plant lectins, by using linear discriminant analysis (LDA). This method enables multiplexed data (*e.g*. like a barcode) to be used as a training matrix to enable prediction of unknown samples, and has been widely used by Rotello *et al*. for non-carbohydrate interactions.^43^ Gibson *et al*. have demonstrated that LDA can be used to distinguish between lectins^18^,^44^ and bacteria^45^ with very similar binding specificities using a minimal number of sugars. However, the key challenge in any sensor is to function across a range of concentrations of analyte (*i.e*. representative of a real system) and potentially give a concentration output, which has not been achieved thus far for lectins. To achieve this with LDA a larger set up of sensory inputs is required and this could be achieved through the use of glycoAuNPs with mixed surface densities, this would add information without increasing the number of glycans (*i.e*. less synthesis required) needed for classification.

Here, glycoAuNPs with heterogeneous coatings of just two monosaccharides are employed to provide discrimination between a small panel of legume lectins using linear discriminant analysis at a range of lectin concentrations. It is shown that the additional complexity introduced by these surfaces increases discrimination power without the need for complex and expensive oligosaccharides. At higher lectin concentration, lectin concentration can also be extracted as well as lectin identity.

## Results and discussion

**Scheme 1:**
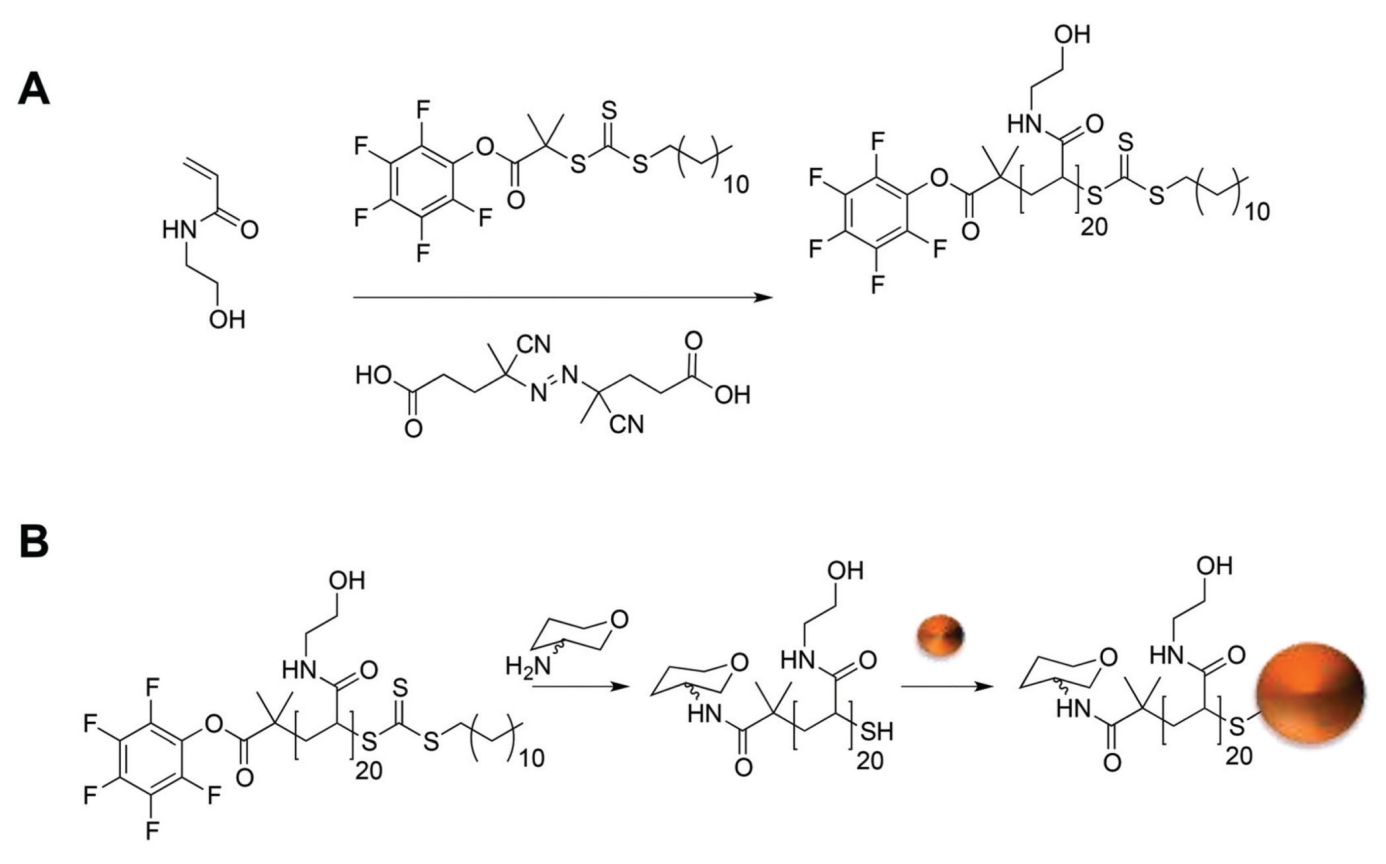
Synthetic route to glycosylated gold nanoparticles: (A) polymerisation of *N*-hydroxyethyl acrylamide using a pentafluorophenyl functional RAFT agent (B) post-polymerisation of pHEA with amino-sugars and immobilisation of carbohydrate terminal polymers onto preformed 60 nm gold nanoparticles.

Inspired by the glycocalyx on cell surfaces, which are heterogeneous, the effect of carbohydrate density on lectin–carbohydrate interaction was determined. Previous studies have shown that by lowering the carbohydrate density on a multivalent scaffold can increase the affinity to the target lectin.^11^,^14^,^40^,^46^,^47^ GlycoAuNPs were prepared using an optimised polymer coating method developed recently by Richards *et al*.^16^ that produces particles that are stable at physiological salt concentration but still result in fast lectin detection, Scheme 1. First, *N*-hydroxyethyl acrylamide (HEA) was polymerised to a degree of polymerisation (DP) of 20 using reversible addition–fragmentation chain transfer (RAFT) polymerisation mediated by a pentafluorophenol α-terminated RAFT agent (Scheme 1A). Then functionalised using 2-amino-2-deoxy-sugars. Functionalisation was confirmed by ^19^F NMR which showed the complete loss of the PFP end group upon addition of the 2-amino-2-deoxy-sugar (ESI†). The carbohydrate-functionalised polymers were subsequently conjugated onto preformed 60 nm AuNPs using the ω-terminal thiol from the RAFT agent (Scheme 1B). Successful polymer coating was demonstrated by XPS analysis (ESI†). Eleven glycoAuNP combinations were prepared, ranging from 100% galactosamine (GalNH_2_) to 100% mannosamine (ManNH_2_) in steps of 10%. The post-polymerisation route employed means that all the polymers have the same initial chain length distribution and therefore reduced the variability between particle types, but allows versatile end-group functionalisation to make libraries of a variety of particles with differing carbohydrate densities.

**Fig. 1:**
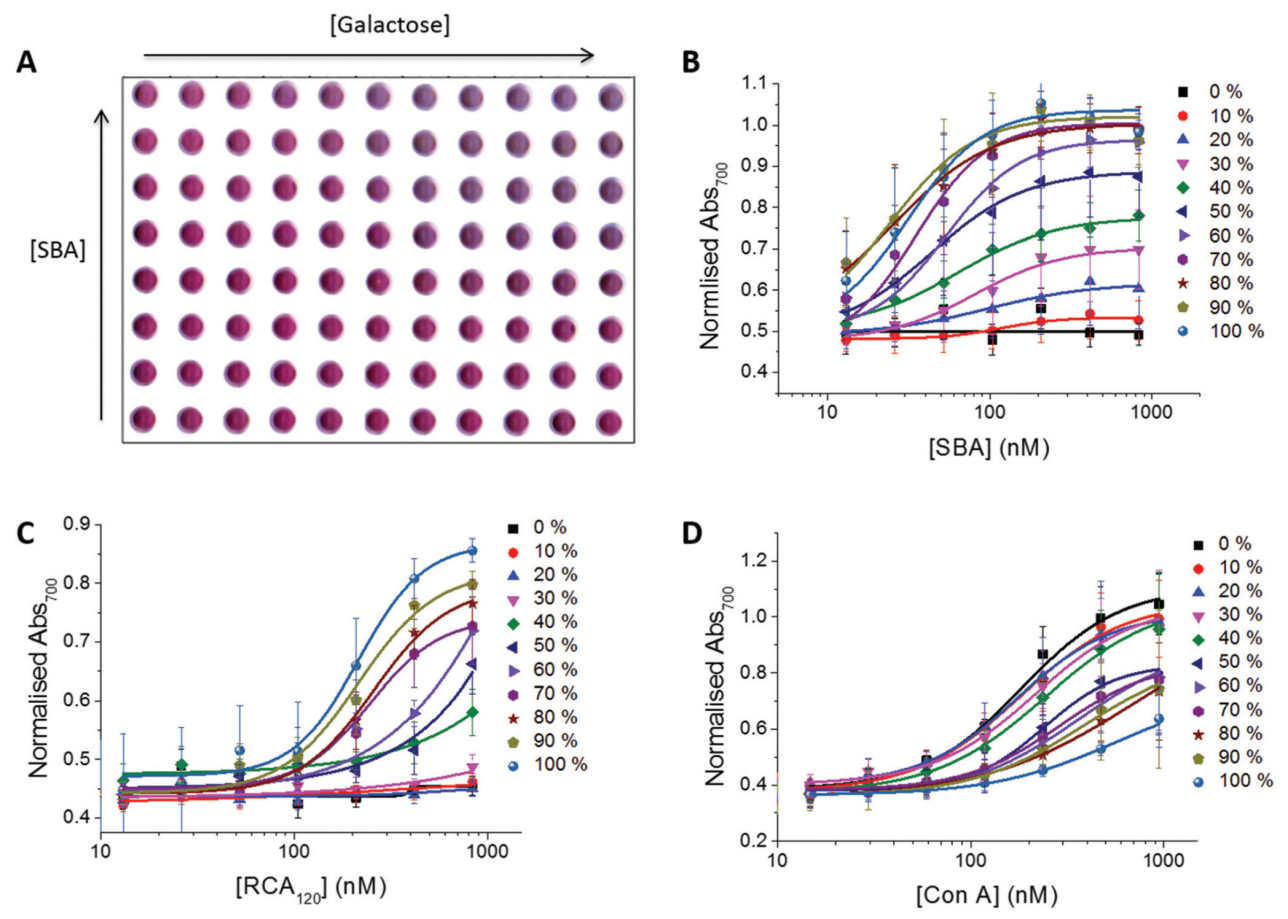
Analysis of lectin–glycoAuNP interactions. (A) Photograph of 96-well plate (background removed) with 11 different glycosylated gold nanoparticles from 100% mannosamine functionality to 100% galactose functionality after incubation with a serial dilution of SBA. Dose-dependent binding isotherms of each glycoAuNP with (B) SBA (C) RCA_120_ (D) Con A.

A serial dilution of each lectin, concavanalin A (Con A), soybean agglutinin (SBA) and *Ricinus communis* agglutinin (RCA_120_ – a model for ricin) were incubated with each glycoAuNP type for 30 minutes and the absorbance spectra measured. A 30 minute incubation period was deemed optimum as previously this interaction has shown to reach steady state after 15 minutes.^16^ Upon aggregation of the particles a colour change from red to blue is clearly seen (an example 96 well plate is shown in Fig. 1A). This change is noted in the UV-vis spectra as a decrease in absorbance at *λ*_max_ and an increase at 700 nm. Dose-dependant binding isotherms for each lectin–glycoAuNP were constructed (Fig. 1B). It should be noted that obtaining this level of data (33 different sets of isotherms) using conventional methods, such as surface plasmon resonance (SPR), isothermal titration calorimetry (ITC) or NMR *etc*. would take a lot of resources and be extremely time consuming, relative to the method presented here.

From plotting the Hill functions of each lectin–glycoAuNP combination, the apparent dissociation constants (*K*_d__app_) could be extracted (Table 1). The selectivity of each lectin towards each monosaccharide was as to be expected and agreed well with microarray data from the Consortium for Functional Glycomics (CFG) database.^47^ For example, SBA is *N*-acetylgalactosamine specific and shows the highest affinity to galactosamine functional particles and Con A is mannose specific and shows the highest affinity for mannosamine functional particles. Fig. 1B shows that below 30% galactosamine content (70%–100% mannosamine) no affinity for SBA is observed. As the galactosamine content is progressively increased above this amount the affinity increases until 70% galactosamine content where no futher increase in affinity is observed. For RCA_120_ at least 70% galactosamine incorporation is required, whereas inclusion of galactosamine is tolerated to a much greater extent with Con A. Control experiments with BSA (a non-carbohydrate binding protein), and an alcohol terminated-polymer coated nanoparticle with the panel of lectins showed no colour or spectral changes confirming that this was a specific interaction not just colloidal instability. These observations highlight that complex heterogeneous glyco-surfaces are a powerful tool to modulate affinity and selectivity, in a manner far more accessible than the total synthesis of complex glycans and hence can be used as the basis of our biosensor.

### Identification of lectins

**Fig. 2:**
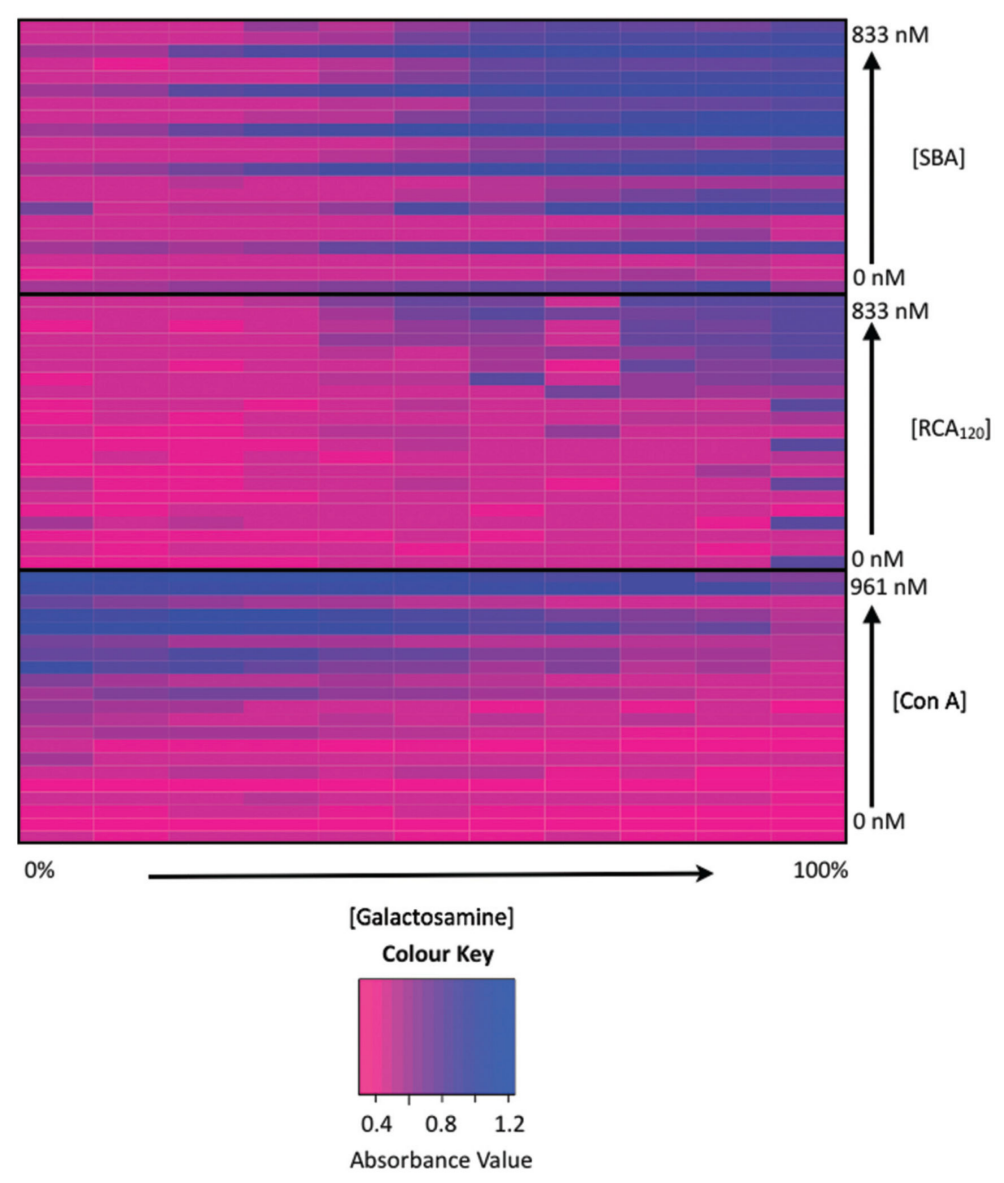
Heatmap showing changes in binding between the lectins at varying concentrations (0–1 μM) to the particles, where blue indicates binding and pink indicates no binding.

Due to lower specificities of lectins towards monosaccharides, it is not always easy to differentiate between lectins when using single sugars sensors, as two different lectins could give a response to the same sugar.^18^ There is also the challenge of differentiating lectins of unknown concentration, with higher concentrations (in our experience) giving better discrimination than lower. To address this, statistical techniques such as linear discriminant analysis (LDA, *vide infra*) can be used to classify analytes with much higher accuracy. Fig. 2 shows a heat map, to present the binding data of the three lectins used here to all 11 nanoparticles as a function of lectin concentration. Blue represents aggregation and pink represents no change in colour (*i.e*. what is seen visually with the nanoparticles). For each lectin there is a clear ‘barcode’ where the relative response of the collection of nanoparticles is unique to that lectin, but is itself, not easy to use as a tool and analyte identification by comparison to this would be highly challenging.

**Fig. 3:**
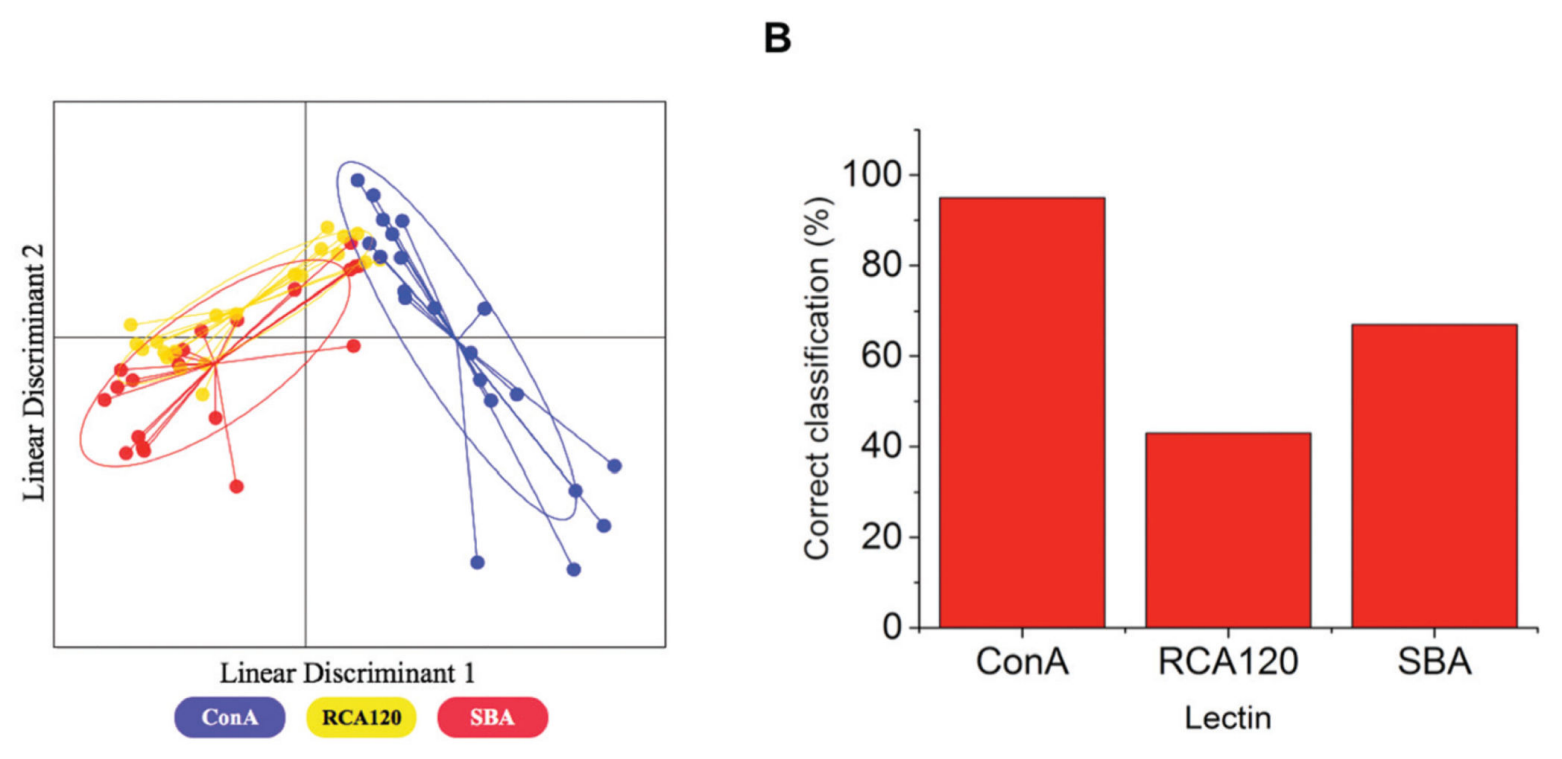
(A) The LDA model generated to discriminate between Con A, RCA_120_ and SBA using binding to only 100% mannose and 100% galactose particles. In the model each point represents a sample of that lectin and the ellipse represents one standard deviation from the average. (B) Correct reassignment percentages of each lectin.

LDA is a powerful machine learning algorithm-based technique that can improve the accuracy of classification of samples to their original groups. It takes an original data set as a training matrix and then generates a model where the variation within the groups (in this case the lectins) is minimised and the variation between groups is maximised in order to achieve the best separation between groups. This then increases the accuracy with which unknown samples can be classified. Production of an LDA model using only the binding profiles of the lectins to 100% mannosamine and 100% galactosamine particles showed significant overlap between RCA_120_ and SBA which would be expected due to their similar binding specificities (Fig. 3A), which in this format would limit application as a sensor and justifying the need for larger training data sets.

This model was then assessed for predictive accuracy in a ‘leave-one-out’ manner, in this case samples were left out of the model at random in an iterative process and then the model that was produced was used to classify the left out samples. Despite the overlap between RCA_120_ and SBA, the model was still able to correctly reassign samples to their lectin classes (in a concentration independent manner) with an accuracy of 68%. The greatest accuracy observed was in the classification of Con A samples and the lowest was that of RCA_120_ with less than half of the samples being correctly classified (Fig. 3B). Furthermore, when this model was used to classify samples based on both lectin class and concentration, it showed a significantly reduced accuracy of 17%. The model was generally better at predicting lectins at higher concentrations and failed to correctly classify any of the lectins at the two lowest concentrations. This serves to highlight the need for larger data sets, to discriminate between more complex analytes, which was investigated in the next steps.

**Fig. 4:**
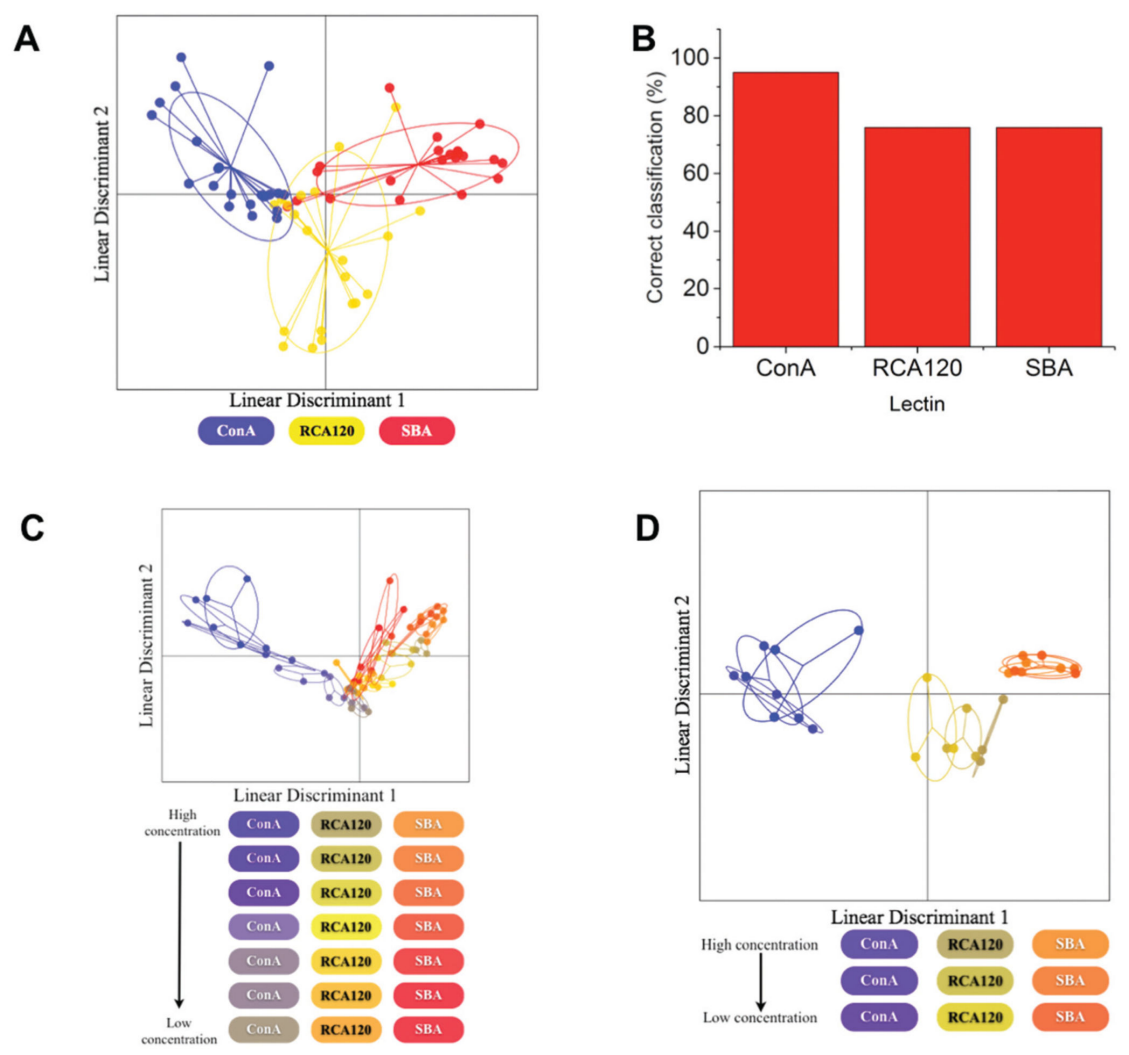
(A) The LDA model generated to discriminate between each lectin, Con A, RCA_120_ and SBA. In the model each point represents a sample of that lectin and the ellipse represents one standard deviation from the average. (B) Correct reassignment percentages of each lectin. (C) The LDA model produced to discriminate between both lectin and concentration. Each points represents a sample of that lectin binding to all of the nanoparticles and the ellipse represents one standard deviation from the average response. (D) The LDA model produced for only those samples where the lectin concentration was >100 nM.

To improve the resolution of the assay, the binding profiles were increased to include binding to all the heterogenous (*i.e*. mixed sugar) particles and the resulting LDA model produced gave clearly much better separation between all of the lectin classes (Fig. 4A). The model showed an increased prediction accuracy and was able to classify samples based on lectin class with an accuracy of 83%. Although little improvement was seen in the classification of Con A samples, the model showed an increase in classification of both SBA and RCA_120_ samples (Fig. 4B). Crucially, the greatest improvement was seen in the classification of samples based on both their lectin and their concentration. The model incorporating all binding data was able to correctly classify both concentration and lectin with an accuracy of 46% (Fig. 4C). Whilst the greatest discrimination was observed at the highest lectin concentrations, as lectins showed good resolution from each other at higher concentrations (Fig. 4D), this model was able to classify some samples correctly even at the lowest lectin concentration. This highlights the true potential of this approach in increasing discriminatory power of a system without increasing the complexity or number of the glycans interrogated as it allowed the identification of lectin class and concentration with an accuracy of 46% and identification of lectin class (independent of sample concentration) with an accuracy of 83% which would be useful for point of care detection of toxins, for example.

## Conclusions

In this study, we have evaluated the use of simple, ratiometric glycosylated gold nanoparticles as low-cost biosensors for lectins and toxin such as ricin. Gold nanoparticles with mixtures of 2 monosaccharides (galactosamine and mannosamine) were employed to generate a label-free assay, which responds to lectin binding by a colour change of red to blue. Generation of the mixed surfaces was combined with a powerful statistical analysis tool (linear discriminant analysis) to generate a model that allowed classification of samples in a concentration-independent way. Addition of mixed surface binding to the model vastly improved the classification of lectins and allowed classification of samples in a concentration-independent manner with an accuracy of 83%. Taken together, this work shows that glycan heterogeneity is a powerful, but synthetically easy, tool to increase complexity and hence increase the discriminatory power of glycan based lectin identification. This will find use in low cost, point of care biosensors, particularly for toxin detection and identification.

## Experimental section

### Materials

All chemicals were used as supplied unless otherwise stated. Acetone, dichloromethane, toluene, methanol, diethyl ether were purchased from Fischer Scientific at laboratory grade. Dodecane thiol (≥98%), potassium phosphate tribasic (≥98%), carbon disulfide (99%), *N*-hydroxethyl acrylamide (97%), 4,4′-azobis(4-cyanovaleric acid) (98%), mesitylene (reagent grade), *N*-(3-dimethylaminopropyl)-*N*′-ethylcarbodiimide hydrochloride (≥98%) were all purchased from Sigma-Aldrich. 2-Bromo-2-methylpropionic acid (98%), 4-(dimethylamino)pyridine (99%), pentafluorophenol (99%), triethylamine (99%) were purchased from Acros. Microtitre plates were purchased from Greiner Bio-one. 10 mmol HEPES buffer containing 0.05 M NaCl, 0.1 mM CaCl_2_ and 0.01 mM MnCl_2_ (pH 7.5, HEPES) was prepared in 200 mL of milliQ water (with a resistance > 19 mOhms). 60 nm gold nanoparticles were obtained from BBI solutions. Concanavalin A and soybean agglutinin and *Ricinus communis* agglutinin-120 were purchased from Vector Labs.

### Physical and analytical methods

NMR spectra were recorded on Bruker DPX-300 and DPX-400 spectrometers for ^1^H NMR (400 MHz) and ^13^C NMR (125 MHz). Chemical shifts are reported in ppm relative to the deuterated solvent resonances and spectra analysed with WIN-NMR software. GPC (DMF) was performed on a Varian 390-LC MDS system equipped with a PL-AS RT/MT auto-sampler, a PL-gel 3 μm (50 × 7.5 mm) guard column, two PL-gel 5 μm (300 × 7.5 mm) mixed-D columns equipped with a differential refractive index, using DMF (with 1 mg per mL LiBr) as the eluent with a flow rate of 1.0 mL min^–1^ at 50 °C. Narrow molecular weight PMMA standards (200–1.0 × 10^6^ g mol^–1^) were used for calibration using a second order polynomial fit. Infrared absorption spectra were recorded on a Bruker VECTOR-22 FTIR spectrometer using a Golden Gate diamond attenuated total reflection cell. Absorbance measurements were recorded on a BioTek Synergy™ multidetection microplate reader using Gen5 1.11 multiple data collection and analysis software.

### Methods

#### End group modification of PFP-polyhydroxyethylacrylamide using 2-amino-2-deoxy-sugars

PFP-pHEA (100 mg, 0.035 mmol), 2-amino-2-deoxy-sugar (3 mg, 5 eq.) were dissolved in 5 mL DMF. The reaction was stirred at 50 °C for 16 h. The polymer was precipitated into diethyl ether from methanol three times and dried under vacuum. IR indicated loss of C==O stretch corresponding to the PFP ester. *M*_n__(theoretical)_ = 2600 g mol^–1^, *M*_n__(sec, dmf)_ = 3700 g mol^–1^, *M*_w (sec, dmf)_ = 4300 g mol^–1^, *M*_w_/*M*_n__(sec, dmf)_ = 1.16.

#### Gold nanoparticle functionalisation using a carbohydrate terminated pHEA

100 μL of 10 mg per mL polymer solution was added to 1 mL of 60 nm particles. Left for 30 minutes at room temperature and centrifuged to remove any unattached polymer and resuspended in water. Presence of polymer coating confirmed by XPS. Particle size determined by absorbance *λ*_SPR_ = 536 nm (60 nm) and DLS (65 nm).

#### Lectin induced aggregation studies by absorbance

A 0.1 mg per mL stock solution of the lectin was made in 10 nM HEPES buffer with 0.05 M NaCl, 0.1 mM CaCl_2_ and 0.01 mM MnCl_2_. 25 μL serial dilution was made up in the same buffer in a low volume 96-well micro-titre plate. 25 μL of the glycoAuNP were added to each well. After 30 minutes an absorbance spectrum was recorded from 450 nm–700 nm with 10 nm intervals.

#### Linear discriminant analysis

A training matrix was prepared with every lectin binding to every particle type in triplicate. This was then subjected to a cross-validation step to ensure that any model produced was not over fitted (ESI†) before being subjected to classical linear discriminant analysis using the ‘dapc’ function in the adegenet package (version 1.4-2)^48^ in the open source statistical package R (version 3.1.3).

## Supplementary Material

†Electronic supplementary information (ESI) available. See DOI: 10.1039/c6an00549g

Supporting Information

## Figures and Tables

**Table 1:** Apparent dissociation constant (*K*_d__app_) in nM for each glycoAuNP and lectin combination determined by Abs_700_

Gal : Man ratio (%)	SBA	RCA_120_	Con A
100 : 0	31.3	203.7	576.2
90 : 10	24.2	223.2	381.2
80 : 20	25.7	259.8	404.5
70 : 30	34.9	275.5	347.4
60 : 40	53.7	466.7	356.6
50 : 50	43.6	673.7	339.3
40 : 60	63.6	556.7	243.9
30 : 70	81.2	N/A	215.8
20 : 80	95.6	N/A	183.2
10 : 90	118.3	N/A	180.2
0 : 100	N/A	N/A	173.4
