# Supplementary material for: Glycan heterogeneity on gold nanoparticles increases lectin discrimination capacity in label-free multiplexed bioassays†
Source: Analyst. Author manuscript; Available in PMC 2016 Jul 21. (PMC4934645; doi:10.1039/c6an00549g)
Supplement: Supporting Information [file NIHMS68803-supplement-Supporting_Information.pdf]

Supporting Information for

Glycan Heterogeneity On Gold Nanoparticles Increases Lectin Discrimination Capacity in Label-Free Multiplexed Bioassays

Lucienne Otten[a,b], Denise Vlachou[a], Sarah-Jane Richards[a,b,*] and Matthew I. Gibson[a,b]

[a] Department of Chemistry, University of Warwick, Coventry, CV4 7AL

[b] Warwick Medical School, University of Warwick, Coventry, CV4 7AL

*Corresponding Author Information

E-mail : S-J.Richards@warwick.ac.uk

Additional Figures

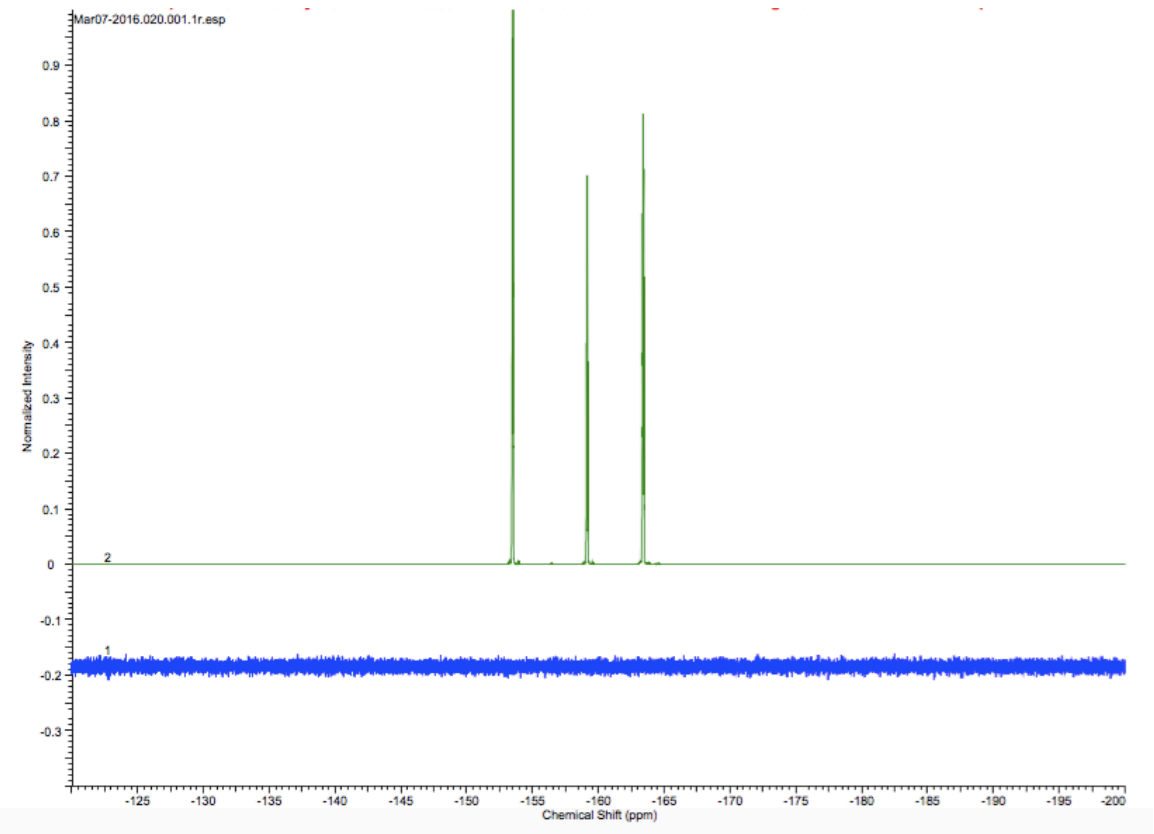

Figure S1: ^{19}F NMR of polymer before (green trace) and after (blue trace) end-group modification.

^{19}F NMR analysis shows presence of PFP-end group before functionalisation and no PFP associated with the polymer after functionalisation with the sugar suggesting 100 % conversion of the end group.

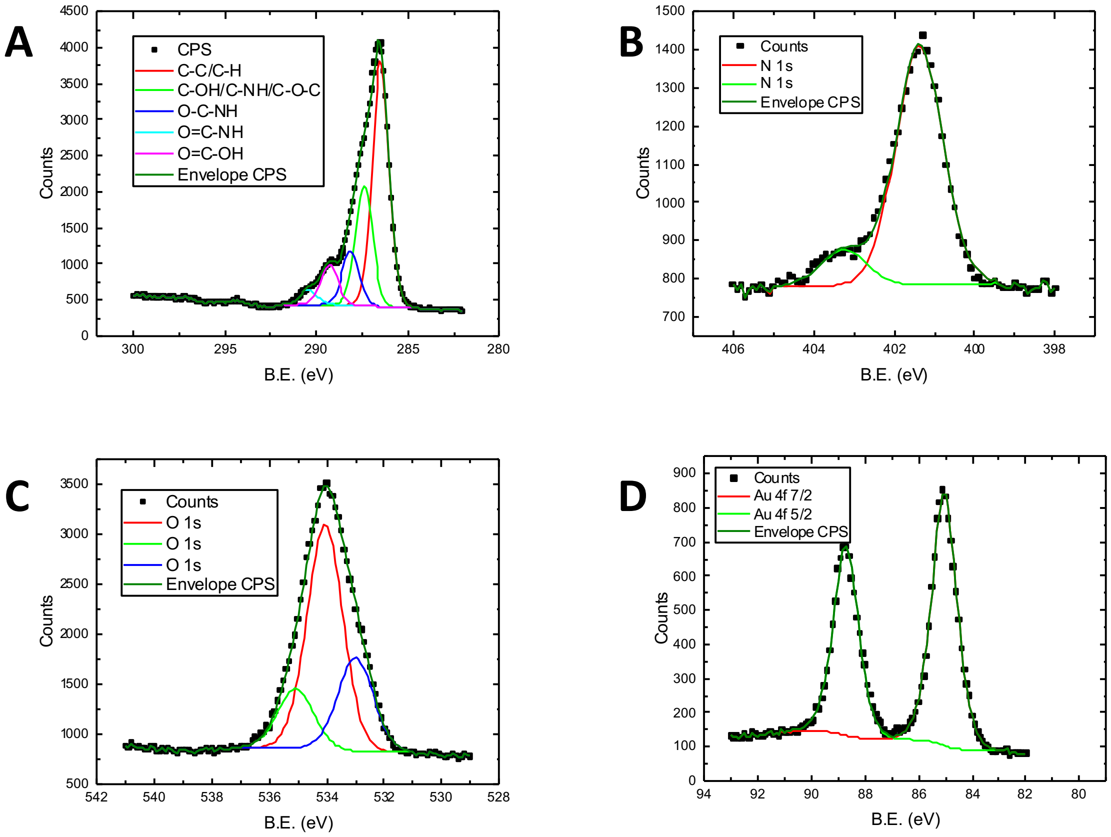

Figure S2: XPS analysis of polymer-coated AuNPs. A) C 1s trace, B) N 1s trace, C) O 1s trace, D) Au 4f trace.

XPS analysis confirms the polymer coating on the particles. The two N 1s correspond to the backbone nitrogen in polymer (red) and the terminal N from the sugar (green).

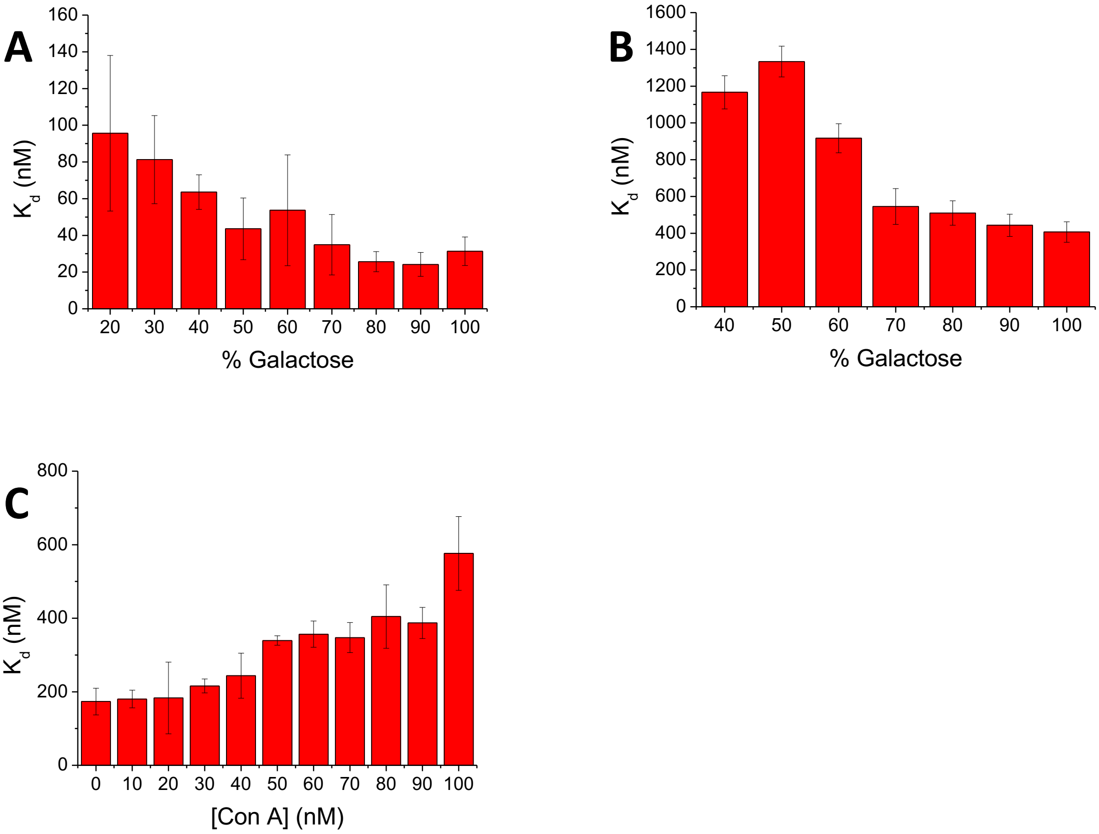

Figure S3: Graphical representation of the K_d values quoted in Table 1 of the main text. A) K_d values for SBA with respect to percentage galactose incorporation, B) K_d values for RCA_{120} with respect to percentage galactose incorporation, C) K_d values for Con A with respect to percentage galactose incorporation.

In order to prevent over fitting of the LDA model, a cross-validation methodology was performed in which a validation data set is predicted from a model based on a training data set. The validation data set and the training data set are randomly selected from the input data and repeated 30 times varying the number of principle components. The darker the blue colour the greater the frequency that outcome occurred. These graphs are then used in order to determine the number of principle components retained to produce the final model. The lowest number of principle components that scored the highest proportion of correct prediction outcomes was retained for each of the models. For the concentration independent data (Figure S4), four principle components were retained and for the concentration dependent data (Figure S5) only two principle components were retained.

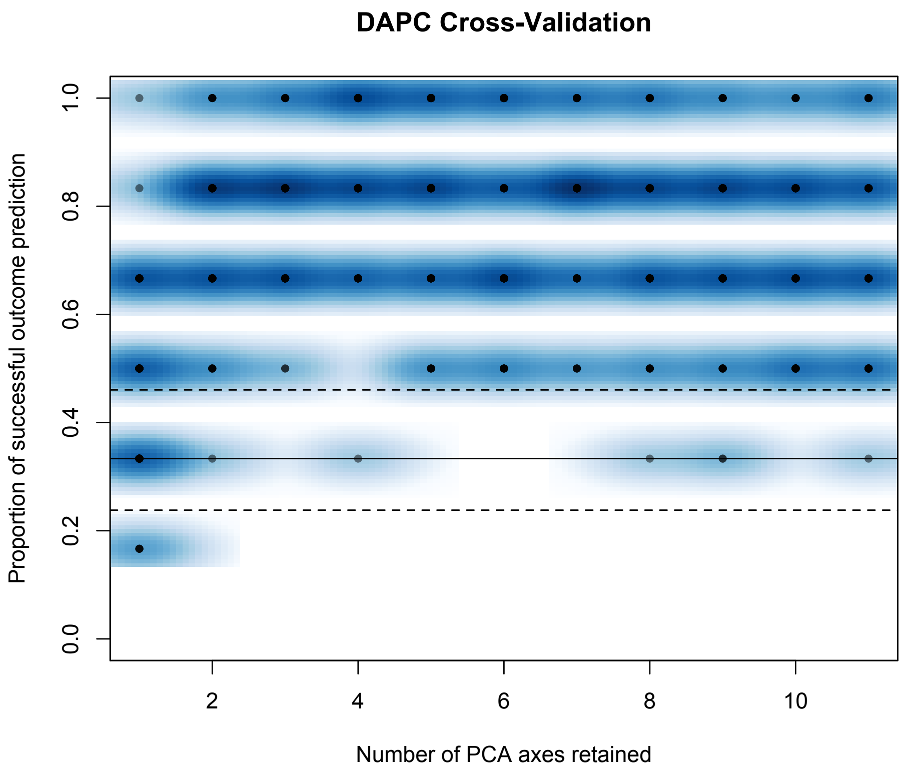

Figure S4: Cross-validation output for concentration independent data

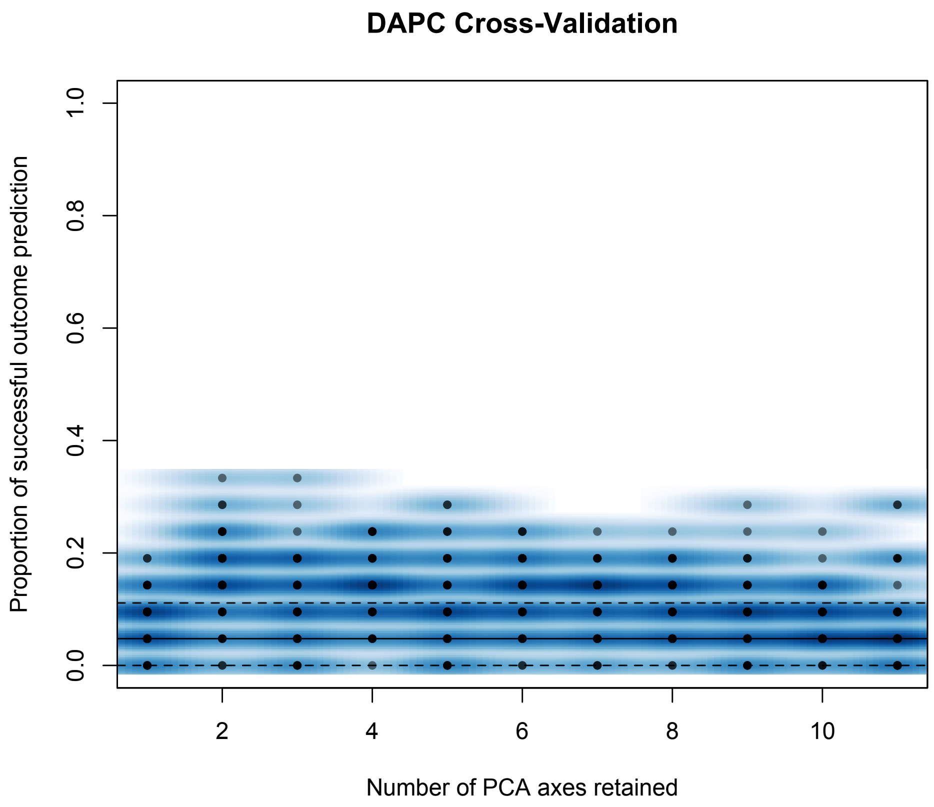

Figure S5: Cross-validation output for concentration independent data
